# Dietary Resource Information for the Oncology Patient: Tips and Tools

**Published:** 2012-01-01

**Authors:** Lydia T. Madsen, Sandra Cesario

**Affiliations:** Ms. Madsen is an advanced practice nurse in the Department of Genitourinary Medical Oncology, The University of Texas MD Anderson Cancer Center, Houston, Texas. Dr. Cesario is a professor and PhD program coordinator at the College of Nursing, Texas Woman's University, Houston, Texas.

Oncology patients frequently request information about diet, exercise, and a healthy living approach during and after cancer treatment. Although a consultation with a registered dietitian is often the recommendation for patients in large multidisciplinary centers, a same-day consult may not always be available. Alternatively, patients might locate diet recommendations by an Internet search, retrieving diet or supplement information that may not follow established optimal health guidelines.

The advanced practitioner is often the most appropriate and available individual able to provide diet and exercise resources and education for patients who request information or require guidance on best practices during treatment. The following information is provided as a concise resource list of books, websites, topics that oncology patients frequently ask about, and briefs that comprehensively address diet and exercise as they pertain to the oncology patient. They each meet the criteria of being readily available to the community and in language that is written for the layperson. There are many additional resources available; this list has been compiled as a small representative sampling of current, scientifically based, patient-directed information to begin or augment a healthful living approach.

**Evidence-based nutrition guidelines for cancer survivors: Current guidelines, knowledge gaps, and future research directions (Robien et al., *Journal of the American Dietetic Association,* 2011)** 

This article from the *Journal of the American Dietetic Association* contains concentrated information that incorporates the current, established guidelines for oncology patients from the American Cancer Society, the American Institute for Cancer Research, and the World Cancer Research Fund. The guidelines are presented in a one-page table format that is easily reproducible as an appropriate, up-to-date handout for patients. An audio podcast of this article is also available online at www.adajournal.org.

The rationale and data supporting diet and lifestyle modifications during and after treatment of the oncology patient presented in this article are compelling; these recommendations are a result of the 2005 Institute of Medicine report calling for a focus on diet and lifestyle modifications in the survivorship population.

One limitation of this article may be that it comes from an evidence-based journal and may contain a higher level of detail than is routinely required by the majority of patients. Statistical reporting may be of limited value to many patients requesting information regarding diet and lifestyle modifications.

***American Cancer Society Complete Guide to Nutrition for Cancer Survivors: Eating Well, Staying Well During and After Cancer* (Grant et al., 2010)** 

Patients are now classified as survivors from the day of diagnosis; this book is therefore appropriate for the entire cancer population. It contains a comprehensive look at all current information as it relates to healthful eating, dietary supplements, the immune system, and lifestyle choices that may enhance cancer survivorship.

This is a well-researched book; supporting data are provided for the information that is presented. Appropriate, relevant information for all patients living with a past or present diagnosis of cancer is contained within the book. Recommendations on management of the challenges associated with the active treatment phase are given in addition to healthy lifestyle recommendations for after treatment. Guidelines for supplements are also provided; this section includes information on the function of each supplement, food sources and daily dietary reference intake details.

Overall, this book is an excellent comprehensive resource for cancer patients and their families.

***Eating Well Through Cancer: Easy Recipes and Recommendations During and After Treatment* (Clegg & Militello, 2006)** 

This book is from the well-known creator of the *Trim & Terrific* cookbook series. The focus is on recipes that are easy to prepare, with the additional benefit of providing nutritional facts essential to patients experiencing side effects related to cancer treatment. The organization of the book is well conceived: the introduction has a recipe cross reference list that contains information related to neutropenia, diarrhea/constipation, high calorie/high protein, sore throat and mouth sores. A nutritional analysis is included with each recipe, in addition to a "Doc’s note" that links the information provided throughout the book with a medical perspective. There is also a section for the caregiver. This is informative and provides valuable suggestions on "what to do to help."

This book is primarily a cookbook; detailed information regarding lifestyle changes is not as extensively discussed as in the other resources in this reference list.

***Nutrition, Exercise and Prostate Cancer. Prostate Cancer Foundation* (Heber et al., 2009)** 

This booklet provides specific, population-targeted information for the prostate cancer patient. It contains an overview of the currently known links between nutrition, exercise, and prostate cancer. There are tips regarding how to best incorporate appropriate nutrition and exercise into daily living. There is also a section on supplement use that provides important information regarding the specific vitamins that can cause toxicity when taken in excess. The booklet can be ordered or downloaded for free (see Reference section for details).

Information regarding the link between obesity and prostate cancer may benefit individuals who seek diet modifications thought to decrease the risk of recurrence. The current references, as suggested readings, provide the scientific basis for the information and are also listed within the booklet.

This booklet may be of limited benefit to individuals seeking information on prostate cancer prevention. The information is provided by an organization that has a fund-raising focus. The suggested reading list contains references that are from medical journals; the articles might contain excessive technical information for the lay population and the articles may be difficult to access.

***Feed Your Hunger for Health and Happiness* (Rosenthal, 2008)** 

This book takes a comprehensive look at how to improve nutrition and health in today’s environment from the complementary perpective. Although this is not a book that specifically targets cancer-related illness, there is a wealth of potentially valuable information to consider regarding the current US culture of fast food, processed foods, and restaurants. There is also an extensive section on recipes that incorporate food items that are not routinely found in a pantry. The recipe section may be beneficial to those individuals hoping to incorporate less familiar food items into a daily diet plan. Examples are given for integrating healthy food items into the diet, such as a simple recipe for hummus as a good, single dietary source for protein, complex carbohydrates, and fiber.

Authored from the complementary perspective, this book may not appeal to patients seeking basic ADA guidelines. The author also has an on-line nutritional counseling program, so there are occasional references to the program that appear to have a marketing slant. The reader may want to consider skipping over those areas to focus on the nutrition and health recommendations with accompanying recipes.

***Anticancer, A New Way of Life* (Servan-Schreiber, 2009)** 

Written by a co-founder of the Center for Integrative Medicine, this book is an informative blend of complementary therapy concepts and known advances in cancer medicine. It also explains recent advances in cancer medicine in understandable vocabulary. A food table is included within the text that provides a listing of specific foods to include in one’s diet to target a healthy lifestyle and decrease the risks of organ-specific cancers. This is a very readable book; extensive referencing for each topic provides a basis for the lifestyle recommendations.

As this book is quite science-based, it may contain a higher level of detail than is routinely requested by patients or family members.

## Internet Website Resources

The following websites were assessed for appropriate, timely information for patients and family members seeking information on cancer and appropriate forms of treatment, with diet and lifestyle recommendations as they pertain to both treatment and survivorship phases. The following criteria were applied and are recommended when selecting accessible Internet resources containing patient education material for patient use:

- The website is published by a recognizable organization, society, or government body. This increases the likelihood that the information is both current and evidence-based.
- The website information is organized in a multidisciplinary format; current appropriate therapies within the website should be included to minimize the risk for bias regarding specific treatment options.
- The website is written for and accessible to the consumer or patient population, rather than solely accessible to health-care providers.
- The website resource list provides patient access to current information in addition to support information on topics of particular interest.
- Statements regarding treatment outcomes can be verified by footnotes or documented research outcome statistics information.

## American Cancer Society (www.cancer.org)

The intended audience for the American Cancer Society (ACS) website is both nonmedical and medical personnel. The website is readily available to consumers; no subscription is required for access. The ACS is a recognized volunteer organization that strives to present both current and evidence-based information specific to a variety of cancers. The educational information provided is written for laypeople in addition to being multidisciplinary; all current treatment options are presented with outcome information related to each type of therapy provided.

There is a tab on the opening page to direct visitors to the *Stay Healthy* section, where current diet, nutrition, and exercise information is provided. The website is routinely maintained so access to this site will contain the most current cancer-specific treatment recommendations.

## MedlinePlus(www.medlineplus.gov)

The MedlinePlus website of the US National Library of Medicine (NLM) through the National Institutes of Health (NIH) is a comprehensive, multidisciplinary directory of cancer information. There are easy-to-navigate tabs on the homepage to take visitors to *Health Topics, Drugs & Supplements*, or *Videos & Cool Tools*. A medical dictionary is provided to define unknown terms, and there are tabs specific to populations with corresponding diseases.

For example, when accessing information on prostate cancer, the directory provides a comprehensive listing of links that are divided into clear subtopics: overviews, latest news, diagnosis/symptoms, screening, treatments, alternative therapies, and nutrition and exercise, to name a few. The directory is easy to navigate; the topics in each cancer subdirectory are well-delineatedand timely; and the resources are clearly labeled and well balanced. Much of the information on MedlinePlus is also available in Spanish.

## Cancer.net (www.cancer.net)

The Cancer.net website is provided by the American Society of Clinical Oncology (ASCO), a nationally prominent societal organization within the field of cancer research and treatment. The website is a comprehensive, multidisciplinary source for cancer information. There are easy-to-navigate tabs located on the home page. When navigating to the section on specific cancers, there is a well-organized, comprehensive listing of links that are divided into subtopics on each cancer: overviews, statistics, diagnosis, prevention and risk factors, treatments, illustrations, clinical trials, current research, and side effects. When looking for either general or organ-specific information about diet and exercise, the search function key is easy to use and produces a wealth of information.

## Healthfinder(www.healthfinder.gov)

This directory website is supported and maintained by the US Department of Health and Human Services. The informational website is well organized and multidisciplinary; various current therapies are listed with detailed descriptions under each cancer site. The website is accessible and available to the consumer population.

No subscription is required for access. The majority of resources are either NIH or other US government publications. This could indicate that the site is less multidisciplinary than other sites, or it could be considered to be less biased; this would depend on the perception of the visitor.

## NCCN (www.nccn.com)

The website for the organization of National Comprehensive Cancer Network (NCCN) is supported as a separate, not-for-profit alliance of 21 of the world’s leading cancer centers, designated as NCI Cancer Centers. The stated mission of the NCCN is a dedication to improving the quality and effectiveness of care provided to people with cancer. The website is easy for patients and family members to access and navigate, and the information provided is extensive and diverse. Patients should be sure to visit www.nccn.com, as opposed to the clinician site www.nccn.org, as the information on the patient site is tailored to that audience.

No subscription is required for access. Upon review of the website's cancer-specific sites, such as breast cancer, the treatments and the patient education sections are multidisciplinary; all current treatment options are presented with current, relevant resource citations. It is possible to search for diet and exercise information either as disease specific or under the heading, "Living with cancer."

## Summary

The preceding information is a sampling of books and websites that were identified to provide current, multidisciplinary information about diet, nutrition, and healthy living as it pertains to the oncology population. The list should be considered a starting point for resource information; it is not a comprehensive listing of resources. Although specific NCI Cancer Center websites were not included because of space limitations, these sites are always excellent resources for current, multidisciplinary treatment, as well as survivorship nutrition and lifestyle recommendations.
